# Parenting Interacts with Oxytocin Polymorphisms to Predict Adolescent Social Anxiety Symptom Development: A Novel Polygenic Approach

**DOI:** 10.1007/s10802-018-0432-8

**Published:** 2018-04-26

**Authors:** Stefanie A. Nelemans, Evelien van Assche, Patricia Bijttebier, Hilde Colpin, Karla van Leeuwen, Karine Verschueren, Stephan Claes, Wim van den Noortgate, Luc Goossens

**Affiliations:** 1 Research Unit School Psychology and Development in Context, KU Leuven, Leuven, Belgium; 2 Research Center Adolescent Development, Utrecht University, PO box 80.140, 3508 TC Utrecht, The Netherlands; 3 GRASP-Research Group, Department of Neuroscience, KU Leuven, Leuven, Belgium; 4 University Psychiatric Center KU Leuven, Leuven, Belgium; 5 Parenting and Special Education Research Group, KU Leuven, Leuven, Belgium; 6 Methodology of Educational Sciences Research Group, KU Leuven, Leuven, Belgium

**Keywords:** Social anxiety symptoms, Adolescence, Oxytocin, Parenting, Gene-by-environment interaction analysis (G × E), Polygenic scores, Longitudinal study

## Abstract

Guided by a developmental psychopathology framework, research has increasingly focused on the interplay of genetics and environment as a predictor of different forms of psychopathology, including social anxiety. In these efforts, the polygenic nature of complex phenotypes such as social anxiety is increasingly recognized, but studies applying polygenic approaches are still scarce. In this study, we applied Principal Covariates Regression as a novel approach to creating polygenic components for the oxytocin system, which has recently been put forward as particularly relevant to social anxiety. Participants were 978 adolescents (49.4% girls; *M*_age_ T_1_ = 13.8 years). Across 3 years, questionnaires were used to assess adolescent social anxiety symptoms and multi-informant reports of parental psychological control and autonomy support. All adolescents were genotyped for 223 oxytocin single nucleotide polymorphisms (SNPs) in 14 genes. Using Principal Covariates Regression, these SNPs could be reduced to five polygenic components. Four components reflected the underlying linkage disequilibrium and ancestry structure, whereas the fifth component, which consisted of small contributions of many SNPs across multiple genes, was strongly positively associated with adolescent social anxiety symptoms, pointing to an index of genetic risk. Moreover, significant interactions were found with this polygenic component and the environmental variables of interest. Specifically, adolescents who scored high on this polygenic component and experienced less adequate parenting (i.e., high psychological control or low autonomy support) showed the highest levels of social anxiety. Implications of these findings are discussed in the context of individual-by-environment models.

**Electronic supplementary material:**

The online version of this article (10.1007/s10802-018-0432-8) contains supplementary material, which is available to authorized users.

## ᅟ

Social anxiety in adolescence has attracted considerable attention in research in the domain of developmental psychopathology and is thought to emerge, in part, through child-by-environment interactions and through the interplay between genetic risk and parenting factors in particular (e.g., Spence and Rapee 2016; Wong and Rapee 2015). Recent progress in the search for genetic main effects (Otowa et al. 2016) has indicated that anxiety has a complex genetic structure. Such findings have caused the field to move from investigating simple, single genetic markers (i.e., candidate genes) to more complex genetic indices that are based on multiple genes (Belsky and Israel 2014; Halldorsdottir and Binder 2016; Nikolova et al. 2011; Purcell et al. 2009). The present study is the first to apply Principal Covariates Regression (De Jong and Kiers 1992; Vervloet et al. 2015), which incorporates elements from factor analysis and regression analysis, in this domain. We used this approach to create composite scores of genetic variation (i.e., polygenic scores) in the oxytocin system related to adolescent social anxiety symptoms that could subsequently be used in interaction analyses with relevant parenting factors to predict social anxiety symptom development from early to mid-adolescence.

### Social Anxiety Symptoms in Adolescence

Adolescence is a critical phase for the development of social anxiety symptoms, which involve a marked and persistent fear of one or more social or performance situations in which the person is exposed to unfamiliar people or to possible scrutiny by others (American Psychiatric Association 2013). Social anxiety symptoms figure among the most prevalent psychopathological symptoms in the general population during adolescence (Kessler et al. 2012), are quite persistent over time, and are associated with a wide range of psychosocial difficulties (Kessler et al. 2012; Kingery et al. 2010; Schneier et al. 1994). Consequently, research that focuses on the development of social anxiety symptoms in adolescence and identifies factors that affect this development is essential.

In recent models of social anxiety, guided by person-by-environment models (Spence and Rapee 2016; Wong and Rapee 2015, 2016), both genetic and environmental factors are thought to play an important role in the development of adolescent social anxiety symptoms. Regarding genetic factors, a recent meta-analysis (Scaini et al. 2014) on data from behavioral genetics (i.e., twin studies) suggested a strong genetic basis for social anxiety in youth (i.e., 53% of the total variance of social anxiety in youth could be explained by the genetic component). Regarding environmental factors, meta-analytic results have also suggested important parental contributions to youths’ social anxiety symptoms (Scaini et al. 2014). Parenting is assumed to play an important role in the development of adolescent social anxiety symptoms. Specifically, low facilitative or high constraining parenting, that is, parenting behavior characterized by overprotection, overcontrol, or rejection (e.g., high psychological control or low autonomy support), has been associated with higher social anxiety symptoms in youth (McLeod et al. 2007; Spence and Rapee 2016; Wong and Rapee 2015, 2016). Overcontrolling and non-autonomy supporting parents tend to be directive and overmanage situations for their adolescents, control and restrict their adolescents’ behaviors and activities, and discourage independence and autonomy in their adolescents. In this way, such parents do not provide adolescents with occasions to explore their environment and develop new and constructive coping and problem-solving strategies for dealing with novel or challenging social situations and may thereby undermine adolescents’ feelings of self-efficacy and increase adolescents’ perception of social threat and reduce adolescents’ perceived control over social threat (Barlow 2002; Rapee 2001; Rubin et al. 2009), which may be associated with greater feelings of social anxiety in adolescents.

### Adolescent Social Anxiety Symptoms: G × E Research

The developmental psychopathology perspective (Cicchetti and Rogosch 2002) recognizes that the development of all forms of psychopathology is affected by multiple risk as well as protective factors at multiple levels of analysis that interact with one another in complex ways over time. Guided by this framework and by person-by-environment models in general, current research efforts increasingly focus on how individual differences in genetic makeup and environmental exposure interact in predicting psychopathological symptoms (i.e., gene-by-environment interactions or G × E), including social anxiety symptoms (e.g., Notzon et al. 2015; Reinelt et al. 2014). These efforts build on the assumption that people vary in the extent to which they are affected by environmental factors and that this sensitivity to the environment may be genetically determined (Pluess 2015). The general expectation is that individuals may be more or less sensitive to environmental factors, both facilitative and constraining, depending on their genetic makeup, which is in turn associated with higher or lower levels of psychopathology.

G × E studies initially adopted a hypothesis-driven approach that is referred to as the *candidate gene approach*, which is based on a single gene, single location principle. This approach is based on the analysis of a single polymorphism (e.g., a variable number of tandem repeats or VNTR, or a single nucleotide polymorphism or SNP) in a particular gene of interest, usually selected for its presumed role in brain function and potential behavioral consequences. Past research on social anxiety has concentrated mostly on genes linked to the serotonin and dopamine signaling pathways (e.g., Enter et al. 2014; Gottschalk and Domschke 2016) because of their presumed role in sensitivity to threat and reward, respectively, which seem relevant to key symptoms of social anxiety, such as heightened sensitivity to social threat and low sensitivity to social reward (Wong and Rapee 2016) as well as heightened reactivity to social stressors (Siess et al. 2014). At the same time, however, sensitivity to threat and reward have been found relevant to a wide range of internalizing and externalizing psychopathological symptoms (Bijttebier et al. 2009), rather than uniquely relevant to social anxiety. In this respect, the oxytocin system has been recently put forward as particularly relevant to social anxiety (e.g., Gottschalk and Domschke 2016; Neumann and Slattery 2016) rather than other forms of psychopathology. Oxytocin has been related to a broad range of complex social behaviors and forms of social cognition and higher levels of oxytocin appear to facilitate social interactions and social approach behavior by reducing reactivity to social stress (Bethlehem et al. 2014). These qualities are clearly relevant to social anxiety symptoms, which are characterized by impaired social interactions and heightened reactivity to social stress (Kingery et al. 2010; Schneier et al. 1994; Siess et al. 2014). Therefore, genes linked to the oxytocin system may be particularly relevant to examine, in interaction with facilitative or constraining parenting, in relation to adolescent social anxiety symptom development.

A limited number of candidate G × E studies on social anxiety symptoms has concentrated on polymorphisms in the oxytocin system and these studies have mainly focused on SNPs in the oxytocin receptor gene (*OXTR*). A significant G × E interaction, for example, has been found between a SNP in *OXTR* (rs2254298) and early adverse parental environment in the prediction of social anxiety symptoms in adolescent girls (Thompson et al. 2011). Specifically, the risk for social anxiety symptoms was highest for those girls exposed to high early adverse parenting and carrying the heterozygote variant of the polymorphism. A significant G × E effect has also been found between another SNP in *OXTR* (rs53576) and attachment style in the prediction of social anxiety symptoms in adults (Notzon et al. 2015). Specifically, the risk for social anxiety symptoms was highest for those individuals with an insecure attachment style and carrying the A-allele of the polymorphism. In sum, genetic variants associated with the oxytocin system and environmental factors have been found to interact in the prediction of social anxiety symptoms.

### From Monogenic to Polygenic Approaches in G × E Research: sG × E

Yet, the use of genome-wide genotyping techniques in *Genome-Wide Association Studies (GWAS)*, which examine thousands of polymorphisms spread across the entire genome (Visscher et al. 2012), has shown that large numbers of polymorphisms are only weakly associated with most phenotypes. Hence, these studies suggest that most types of psychopathology are highly polygenic and that a combination of genetic variants is linked to psychopathology risk (Belsky and Israel 2014; Otowa et al. 2016; Purcell et al. 2009), in concert with the environment. By focusing on a single SNP in a single gene, the candidate gene approach therefore only captures a limited amount of genetic variance. Critical reflections on the candidate gene approach and recommendations for future G × E studies (e.g., Dick et al. 2015) have recently inspired a shift away from monogenic approaches to polygenic approaches that combine information on genetic variation across multiple genes and loci.

As of yet, studies that apply a polygenic approach and also take the environment into account are still scarce (but see e.g., Peyrot et al. 2014; Salvatore et al. 2015; Vrshek-Schallhorn et al. 2015), in research on adolescent social anxiety symptoms in particular. When such polygenic scores that are based on individual differences in the summarized main effects of SNPs on a certain phenotype are used as a variable that interacts with the environment, this approach addresses a slightly different question than traditional G × E candidate gene analyses. Specifically, such *genetic component score-by-environment interaction* (sG × E) analyses—a term we would like to introduce to distinguish such analyses from traditional G × E candidate gene analyses—address the question how the environment that a particular individual is exposed to can alter the risk for a psychiatric disorder, as expressed in a polygenic score.

Polygenic scores for sG × E analyses can be created using either a hypothesis-driven or a hypothesis-free approach (Belsky and Israel 2014). The hypothesis-driven approach, also referred to as *Multilocus Profile Scores* (MPSs), is an extension of the candidate gene approach. In this approach, polygenic scores are created by summing predefined risk alleles (i.e., based on previous candidate gene results) from known polymorphisms in several genes, often belonging to a given biological system (e.g., Nikolova et al. 2011; Vrshek-Schallhorn et al. 2015). The hypothesis-free approach, also known as *Polygenic Risk Scores* (PRSs), has its foundation in GWAS studies. In this approach, the polygenic scores are a sum of alleles from thousands of SNPs across the entire genome, weighted by the estimated strength of the relation between the SNPs and the phenotype (for a review, see Halldorsdottir and Binder 2016; see also Peyrot et al. 2014; Purcell et al. 2009; Salvatore et al. 2015).

### A Novel Polygenic Approach: Principal Covariates Regression

In this study, we introduce an approach that combines aspects of both of these existing polygenic approaches and can be applied to selections of a few hundred SNPs, which is often seen in studies that are set up as an extension of traditional candidate gene studies. As such studies are not GWAS, the guiding principles of the PRS approach do not apply. However, the number of genetic variants in such cases is too large to apply the MPS approach as researchers often do not have prior information on possible risk alleles for all SNPs. Therefore, we introduce a novel data reduction method in this field, that is, Principal Covariates Regression, which allows researchers to estimate the polygenic association of a particular selection of SNPs with a certain phenotype that does not require prior knowledge of genetic risk variants.

Principal Covariates Regression (De Jong and Kiers 1992) reduces predictor variables (in the current study, a few hundred SNPs associated with the oxytocin system) to a limited number of overarching components and at the same time regresses the outcome at hand (in the current study, adolescent social anxiety symptoms) on those components. In other words, the polygenic components are derived in such a way that they summarize the polymorphisms in overarching components, much like factor analysis, while taking into account prediction of the outcome at hand, as is the case in regression analysis. Principal Covariates Regression was developed for use in situations where the number of predictors is large and some of these predictors are highly correlated, in which situations traditional regression techniques may yield unstable results. Although this technique has not been applied before for genetic analysis, it might be particularly suited for this purpose because SNPs can be highly correlated due to the underlying linkage disequilibrium (LD) structure (i.e., non-random association of alleles at different loci). The technique has recently gained in popularity as an R package to conduct Principal Covariates Regression analyses has become available (PCovR; Vervloet et al. 2015).

Importantly, Principal Covariates Regression is different from some of the currently applied data reduction techniques that have been occasionally used to capture polygenic variation, such as Supervised Principal Component Analysis (SPCA; Bair et al. 2006) or gene set analysis (De Leeuw et al. 2015). These techniques are typically employed to elucidate biological mechanisms by searching for specific genes associated with a specific phenotype. In contrast, guided by psychological individual differences research, we employed Principal Covariates Regression to summarize individual differences in genetic variation associated with the oxytocin system in the best possible way in order to include these summarized polygenic component scores in subsequent sG × E analyses. Thus, by employing Principal Covariates Regression we aimed to adequately capture individual differences across hundreds of SNPs in polygenic scores and examine how these polygenic scores interact with environmental exposure to predict adolescent social anxiety symptoms.

### The Present Study

In the present study, we aimed to investigate the interaction between polygenic components comprising genetic variation in the oxytocin system and both constraining and facilitative aspects of parenting, parental psychological control and autonomy support, respectively, in association with social anxiety symptom development across 3 successive years in a large sample of adolescents. To create polygenic components, we applied Principal Covariates Regression as a novel analytical approach. As this represents the first application of this technique to genetic data, we had only global expectations rather than specific hypotheses regarding the genetic components we would find. We expected some polygenic risk components to emerge (i.e., components with a positive regression coefficient) and possibly some protective components (i.e., components with a negative regression coefficient) as well. A multi-informant latent index consisting of adolescent self-reports and both mother- and father-reports of parental psychological control or autonomy support across 3 successive years was included as environmental factor in our sG × E interaction analyses to increase the assessment quality of environmental exposure (Wong et al. 2003). In line with previous studies and theoretical models on the development of social anxiety symptoms (McLeod et al. 2007; Spence and Rapee 2016; Wong and Rapee 2015, 2016), we expected that more parental psychological control would be associated with higher levels of adolescent social anxiety symptoms, whereas more parental autonomy support would be associated with lower levels of adolescent social anxiety symptoms. Finally, regarding the sG × E interactions, in line with the environmental sensitivity framework (Pluess 2015) we expected to find the highest social anxiety symptoms among those adolescents with a high polygenic load and experiences of less adequate parenting (i.e., high parental psychological control or low parental autonomy support).

## Method

### Participants

Participants were 1116 adolescents (49.0% girls; *M*_age_ T_1_ = 13.79 years, *SD*_age_ T_1_ = 0.94) who took part in the ongoing longitudinal “Studying Transactions in Adolescence: Testing Genes in Interaction with Environments” (STRATEGIES) study in Flanders, the Dutch-speaking part of Belgium. All participants attended Grades 7 to 9 at the start of the study and completed the questionnaires three times with a one-year interval between successive waves. The majority lived in intact two-parent families (79.2%). For the present study, participants’ family structure was dichotomized into (0) living in intact two-parent families or (1) other family structure.

A total of 1103 participants were genotyped (98.8% response rate). Following quality control (see Online Supplementary Material 1), the dataset comprised of 1031 participants. Of those, 978 participants reported on social anxiety symptoms at T_1_ (49.4% girls; *M*_age_ T_1_ = 13.80 years, *SD*_age_ T_1_ = 0.94). There were no significant differences between the total sample (*N* = 1116) and our study sample including participants with high-quality genetic data and data on social anxiety symptoms (*n* = 978) regarding sex, χ^2^(1) = 0.56, *p* = 0.46, age, *F*(1, 1093) = 0.22, *p* = 0.64, grade level, χ^2^(2) = 0.97, *p* = 0.61, and family structure, χ^2^(2) = 0.01, *p* = 0.94.

### Procedure

Data collection for the STRATEGIES study started in February–March 2012 in nine secondary schools in Flanders. A randomized multistage sampling approach was used to select participants. Several Flemish secondary schools from different provinces were invited to take part in the research project, stratified by educational track in order to include participants from the academic, technical, and vocational tracks. From the nine schools that were willing to participate, 121 classes from Grades 7 to 9 were randomly selected to participate. Within these classes, all adolescents were invited to participate. Active written informed consent was obtained from both parents and adolescents before the start of the study (*N* = 1116). At each wave, participants completed questionnaires in a 50-min session in their classroom during regular school time. Research assistants supervised these sessions and provided instructions, ensured confidentiality, and answered questions when necessary. This study received ethical approval from the Biomedical Institutional Review Board at the KU Leuven, Belgium.

DNA was obtained from saliva samples, which were collected at the first measurement occasion using the Oragene^®^ DNA collection kits (DNA Genotek). SNPs were selected using a step-wise procedure. First, an extensive literature search was conducted for “usual suspects”, that is, candidate genes and their SNPs that had already been associated repeatedly with social anxiety symptoms or related psychological constructs (e.g., depressive symptoms). These included, among others, the SNPs rs53576 and rs2254298 in the *OXTR* gene that were discussed in the Introduction. Second, we included SNPs from genes that showed a high degree of connectedness to these candidate genes, based on known protein databases (e.g., STRING; Szklarczyk et al. 2011). Third, for each of the genes selected in the previous two steps and the regions closely surrounding them, additional so-called tagging SNPs were selected. Using Haploview (Barrett et al. 2005), these additional SNPs were selected in a way that they would cover at least 80% of the variability in each gene while taking into account LD norms (i.e., SNPs were evenly distributed across each gene).

SNPs were analyzed using an Illumina Infinium iSelect Custom BeadChip. The DNA samples were processed according to the protocols provided by Illumina (Illumina 2011) and genotyped using Illumina iScan (Illumina 2010). The protocols of Anderson et al. (2010) and Purcell et al. (2009) were used for quality control (see Online Supplementary Material 1 for details) and Beagle genetic analysis software was used to impute the few missing allele frequencies (based on 95% call-rate). In this study, we only selected genes associated with the oxytocin pathway (see Ebstein et al. 2012), which led to a total of 223 oxytocin SNPs situated in 14 genes (see Table S1 in Online Supplementary Material 1).

### Measures

#### Social Anxiety Symptoms

We used a 12-item short version of the Social Anxiety Scale for Adolescents (SAS-A; La Greca and Lopez 1998) to assess adolescents’ social anxiety symptoms. The SAS-A consists of three subscales: Fear of Negative Evaluation, Social Avoidance and Distress to New Situations, and Generalized Social Avoidance and Distress. The 12-item short version of the SAS-A consists of the four highest loading items for each subscale that have been consistently found to load substantially on their designated factor in previous studies (see Nelemans et al. 2017). Sample items include “I worry about what others say about me”, “I feel shy around people I don’t know”, and “I am quiet when I’m with a group of people”, respectively. All items were rated on a 5-point Likert-type scale, ranging from 1 (*not at all*) to 5 (*all the time*). We found good internal consistency for the total SAS-A scale across all 3 years (Cronbach’s α = 0.92). Higher total SAS-A scores reflect higher levels of social anxiety.

#### Parental Psychological Control and Autonomy Support

We used the 9-item psychological control and 8-item autonomy support subscales described in Janssens et al. (2015). These subscales are Dutch adaptations of well-established instruments originally developed in the United States, that is, the Psychological Control Scale – Youth Self-Report (PRS-YSR; Barber 2002) and the Perceptions of Parents Scale (POPS; Grolnick et al. 1991), respectively. Adolescents reported on their perception of parental psychological control and autonomy support and both mothers and fathers reported on their perception of their own parental psychological control and autonomy support across 3 successive years. Sample items include “My parents will avoid looking at me when I have disappointed them” for adolescent-reported parental psychological control and “I help my son/daughter to choose his/her own direction” for mother/father-reported parental autonomy support. All items were rated on a 5-point Likert-type scale, ranging from 1 (*completely disagree*) to 5 (*completely agree*). We found good internal consistency for both the psychological control subscale (Cronbach’s α = 0.71–0.83) and the autonomy support subscale (Cronbach’s α = 0.80–0.87) for all informants across all 3 years. Higher scores reflect higher levels of parental psychological control and autonomy support and moderate positive concurrent associations were found among reports of different informants on parental psychological control, *r* = 0.15–0.44, and parental autonomy support, *r* = 0.18–0.40, across 3 years.

### Statistical Analyses

First, we used Principal Covariates Regression (De Jong and Kiers 1992) to create polygenic components based on our 223 oxytocin SNPs. For this purpose, we applied the R package PCovR (Vervloet et al. 2015) to our 223 oxytocin SNPs with adolescent social anxiety symptoms at T_1_ as outcome variable. PCovR is a flexible analytical tool that allows the user to (a) choose the extent to which summarizing the SNPs and prediction of the outcome play a role when constructing the polygenic components, (b) predefine a minimal, maximum, or exact number of components to summarize the SNPs, and (c) select different orthogonal and oblique rotation strategies for interpreting the components. In general, model selection in PCovR thus involves decisions in these three areas. Optimizing the PCovR solution was achieved through the selection of the optimal weighing parameter, the α value (Vervloet et al. 2015). Higher α values (i.e., closer to 1) assign a greater role to reduction of the predictor variables when constructing the components and lower α values (i.e., closer to 0) assign a greater role to prediction of the outcome variable when constructing the components. Given the known genetic interdependency between SNPs, as reflected by their LD structure, we expected to find some genetic components mirroring the LD structure with α values close to 1 (see Online Supplementary Material 1).

We used a manual sequential approach for choosing the best PCovR model. Specifically, we searched for an optimal balance between reduction of the genetic information and prediction of adolescent social anxiety symptoms by looking at changes in explained variance in both with slight changes in the α value (see Figure S1 in Online Supplementary Material 1). After selecting the PCovR model with the best balance between reduction of the oxytocin SNPs and prediction of adolescent social anxiety symptoms, we used the quartimin rotation strategy for interpretation of the polygenic components (i.e., an oblique rotation strategy that allows for correlated components).

Subsequently, we used participants’ scores on all polygenic components that did not merely reflect the LD structure but had substantive meaning in relation to adolescent social anxiety symptoms, in our interaction analyses in M*plus* Version 7.4 (Muthén and Muthén 1998-2015). To model development of adolescent social anxiety symptoms, we set up a Latent Growth Curve Model with initial levels of social anxiety symptoms (i.e., intercept) and linear change in these symptoms across 3 years (i.e., slope). Also, in M*plus* we created a latent factor of all informant reports on either parental psychological control or parental autonomy support across 3 years and had this latent index of parenting interact with participants’ polygenic component scores. We standardized all genetic components and our latent indices of parenting before creating interaction terms. Analyses were conducted separately for parental psychological control and parental autonomy support and all analyses included adolescents’ sex, age, and family structure as potential covariates, all correlations among the polygenic components, and correlations between the polygenic components and the latent indices of parenting to account for potential gene-environment correlation. We used ML estimation with standard errors and chi-square robust to non-normality (i.e., MLR estimator; Muthén and Muthén 1998-2015).

## Results

### Descriptive Statistics

Means and standard deviations of all study variables across all 3 years are shown in Table S2 (see Online Supplementary Material 1). Concurrent associations were weak to moderate between adolescent social anxiety symptoms and reports of parental psychological control, *r* = 0.01–0.23, and weak between adolescent social anxiety symptoms and reports of parental autonomy support, *r* = -0.11 – 0.04, across informants across 3 years.

### Principal Covariates Regression

First, we explored PCovR results for different α values ranging from 0 to 1. Specifically, we explored the full range of α values between 0 and 1 in steps of 0.10 to get an idea of the optimal α value (i.e., the optimal balance between reduction of the oxytocin SNPs and prediction of adolescent social anxiety symptoms). As large changes in explained variance appeared to occur between α values of 0.70 and 1.00, we conducted a more in-depth exploration of PCovR results across this range of values in steps of 0.01. These results are represented graphically in Figure S1 (Online Supplementary Material 1).

An α value of 0.80 appeared to show the best balance between reduction of the oxytocin SNPs and prediction of adolescent social anxiety symptoms. At this α value, the highest level of variance was explained in adolescent social anxiety symptoms while at the same time still a large amount of variance was explained in the 223 oxytocin SNPs by the five resulting genetic components in this PCovR solution (see Figure S1 in Online Supplementary Material 1). In this PCovR solution, one polygenic component was strongly positively associated with adolescent social anxiety symptoms, β = 0.49, 95% CI [0.44, 0.54], *p* < 0.001. This polygenic component appeared to be spread across the 223 oxytocin SNPs with a few factor loadings showing medium effect sizes (i.e., ≥ 0.15 in absolute value; Cohen 1992). Those SNPs with medium effect sizes were located on Chromosome 5 in the prolactin receptor gene (*PRLR*) and the gamma-aminobutyric acid receptor alpha-6 subunit gene (*GABRA6*), and on Chromosome 3 in the oxytocin receptor gene (*OXTR)*.

The other four components appeared to strongly reflect the underlying linkage disequilibrium (LD) structure, as anticipated (see Online Supplementary Material 1 and Figure S2), and contained information on population stratification, which refers to individual differences in genetic variants due to individual differences in ancestry or ethnicity (see Online Supplementary Material 1). Even though the substantive interpretation of these four components is limited in relation to adolescent social anxiety symptoms, these genetic components do serve as a form of validation for the use of PCovR with genetic data.

#### Auxiliary Analyses

Several additional analyses were conducted to examine the robustness and specificity of our polygenic findings (see Online Supplementary Material 1).

#### Robustness

To examine the robustness of our findings, we examined how several factors affected the polygenic components or associations between the polygenic components and adolescent social anxiety symptoms (see Online Supplementary Material 1). There were no significant associations between adolescents’ age, sex, or family structure and the polygenic components and including adolescents’ age, sex, and family structure as covariates did not change the predictive values (i.e., βs) of the components on adolescent social anxiety symptoms. Also, adolescents’ sex did not significantly moderate any of the associations between the components and adolescent social anxiety symptoms. Finally, randomly deleting 1%, 5%, or 10% of the oxytocin SNPs (i.e., 2, 11, or 22 SNPs out of the 223 oxytocin SNPs, respectively) from the PCovR analyses did not substantially affect the composition of the five polygenic components nor the predictive values of these components for adolescent social anxiety symptoms. Collectively, these results corroborated the robustness of our PCovR findings.

#### Specificity

We examined associations between the polygenic components and broader adolescent psychological functioning (i.e., adolescent internalizing problems other than social anxiety symptoms and adolescent externalizing problem behaviors; see Online Supplementary Material 1). The components appeared to be more strongly associated with adolescent social anxiety symptoms than adolescent depressive symptoms, feelings of loneliness, levels of neuroticism, and externalizing symptoms, which corroborated the specificity of our findings.

### sG × E Interaction Analyses^1^

**Fig. 1:**
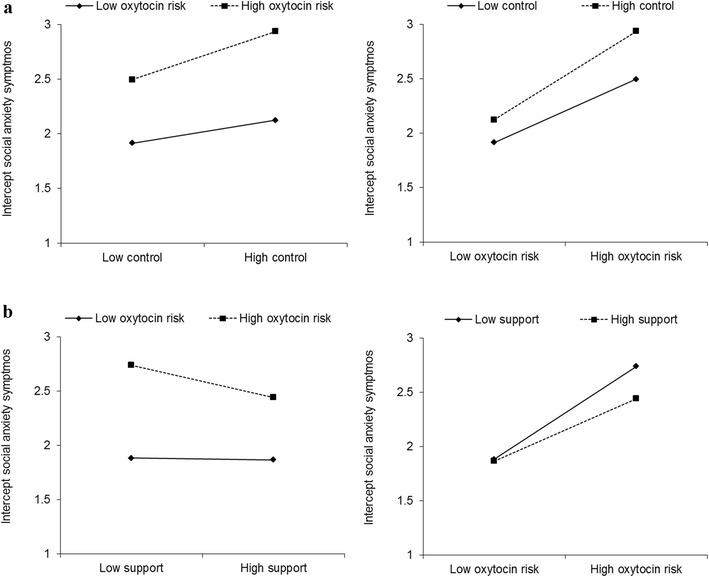
Graphic representation of the significant G × E interactions involving polygenic risk in the oxytocin system and a latent index of parental psychological control (**a**) and involving polygenic risk in the oxytocin system and a latent index of parental autonomy support (**b**) predicting adolescent social anxiety symptoms for high (+1 SD) and low (−1 SD) values. Control = parental psychological control. Support = parental autonomy support. Adolescent social anxiety symptoms range from 1 to 5 (mean levels)

Results from our sG × E interaction analyses are shown in Table 1. These analyses included controls for gene-environment correlations, which ranged from −0.03 to 0.07 (all *n.s.*) for parental psychological control and from −0.02 to −0.08 (all *n.s.*) for parental autonomy support. In line with our expectations, results suggested that higher parental psychological control, β = 0.24, 95% CI [0.16, 0.32], *p* < 0.001, and lower parental autonomy support, β = −0.12, 95% CI [−0.04, −0.20], *p* = 0.004, were significantly associated with higher initial/intercept levels of social anxiety. Moreover, the polygenic oxytocin component that was substantively associated with adolescent social anxiety symptoms (consisting of small contributions of many SNPs across multiple genes) showed a significant interaction with parenting in predicting adolescent social anxiety symptom development. Specifically, results suggested a significant interaction between this polygenic component, on the one hand, and our latent indices of parental psychological control, β = 0.09, 95% CI [0.03, 0.15], *p* = 0.004, and parental autonomy support, β = −0.10, 95% CI [−0.04, −0.17], *p* = 0.001, on the other hand, on initial/intercept levels of adolescent social anxiety. These interactions are represented graphically in Fig. 1. Both interactions were in line with our expectations: The highest levels of social anxiety were reported by those adolescents with higher scores on the polygenic component and experiences of higher levels of parental psychological control or lower levels of parental autonomy support. In contrast, those adolescents with lower scores on the polygenic component consistently showed the lowest levels of social anxiety, regardless of the parenting context. In sum, adolescents’ polygenic oxytocin component score interacted with both constraining and facilitative aspects of parenting to predict intercept levels of social anxiety.

**Table 1:** Structural equation models predicting adolescent social anxiety symptom development across 3 successive years (*N* = 973)

	Parental psychological control				Parental autonomy support			
	Intercept		Linear slope		Intercept		Linear slope	
Predictor	*b (SE)*	β	*b (SE)*	β	*b (SE)*	β	*b (SE)*	β
Parenting	0.16 (0.03)^***^	0.24	0.01 (0.02)	0.04	−0.08 (0.03)^**^	−0.12	−0.00 (0.02)	−0.01
Polygenic C1	0.35 (0.02)^***^	0.52	−0.08 (0.01)^***^	−0.27	0.36 (0.02)^***^	0.53	−0.07 (0.01)^***^	−0.26
Polygenic C2	−0.13 (0.02)^***^	−0.19	0.01 (0.01)	0.03	−0.14 (0.02)^***^	−0.20	0.01 (0.01)	0.04
Polygenic C3	0.04 (0.02)^*^	0.06	−0.01 (0.01)	−0.02	0.03 (0.02)	0.05	−0.01 (0.01)	−0.02
Polygenic C4	−0.02 (0.02)	−0.02	−0.01 (0.01)	−0.02	−0.01 (0.02)	−0.02	−0.01 (0.01)	−0.02
Polygenic C5	0.00 (0.02)	0.01	−0.01 (0.01)	−0.02	0.00 (0.02)	0.00	−0.01 (0.01)	−0.03
Parenting × C1	0.06 (0.02)^**^	0.09	−0.04 (0.02)^**^	−0.16	−0.07 (0.02)^***^	−0.10	0.03 (0.02)^†^	0.10
Sex	0.23 (0.04)^***^	0.34	0.06 (0.03)^*^	0.20	0.22 (0.04)^***^	0.33	0.06 (0.03)^*^	0.20
Age	−0.00 (0.02)	−0.01	−0.01 (0.01)	−0.05	0.01 (0.02)	0.01	−0.01 (0.01)	−0.05
Family structure	−0.08 (0.05)	−0.12	0.04 (0.03)	0.12	−0.08 (0.05)	−0.12	0.04 (0.03)	0.13

C1-C5 represent the five polygenic oxytocin components resulting from the PCovR analysis. Sex was coded 0 for boys and 1 for girls and family structure was coded 0 for living in intact two-parent families and 1 for other family structure

^†^*p* ≤ 0.08. ^*^p ≤ 0.05. ^**^p ≤ 0.01. ^***^p ≤ 0.001

In addition, we found a significant interaction between this polygenic component and our latent index of parental psychological control, β = −0.16, 95% CI [−0.04, −0.27], *p* = 0.007, as well as a marginally significant interaction with our latent index of parental autonomy support, β = 0.10, 95% CI [−0.01, 0.22], *p* = 0.073, on change in levels of adolescent social anxiety across 3 years. Combined with the findings mentioned earlier, these interactions suggest that adolescents with the highest initial/intercept levels of social anxiety (i.e., those with higher polygenic component scores and higher parental psychological control or lower parental autonomy support) reported the smallest increase in social anxiety symptoms. In contrast, those adolescents with the lowest intercept levels of social anxiety (i.e., those with lower polygenic component scores) reported a stronger increase in social anxiety symptoms, particularly in a constraining parenting context (i.e., high parental psychological control or low parental autonomy support).

Importantly, these significant sG × E interactions were found including gender as predictor of both the intercept and the slope in our models. Results suggested that girls reported both higher initial levels of social anxiety symptoms, *b =* 0.22–0.23, β = 0.33–0.34, *p* < 0.001, and a stronger increase in social anxiety symptoms over time, *b =* 0.06, β = 0.20, *p* = 0.03, than boys. This pattern of findings suggests that the sG × E interactions explained significant variance in adolescent social anxiety symptom development in addition to the gender differences just mentioned.

#### Auxiliary Analyses

Several additional analyses were conducted to examine the robustness and sensitivity of our sG × E findings.

#### Robustness

We conducted additional analyses in which we included quadratic terms for both the polygenic component and either parental psychological control or parental autonomy support in addition to the sG × E interaction, because the sG × E interaction can carry the variance of the unmodeled quadratic terms and generate spurious interactions (see Dick et al. 2015). Including these quadratic terms in our models did not affect any of our conclusions: The results still suggested a significant interaction between the polygenic component and our latent indices of parental psychological control and parental autonomy support on initial/intercept levels of adolescent social anxiety, β = 0.09, *p* = 0.007 and β = −0.10, *p* = 0.002, respectively, as well as a significant interaction between this polygenic component and parental psychological control on change in levels of adolescent social anxiety across 3 years, β = −0.17, *p* = 0.010. This result suggests that the significant sG × E interactions are unlikely to be spurious.

#### Sensitivity

We conducted additional analyses to examine the robustness of our sG × E findings across informants (see Online Supplementary Material 1). Importantly, our sG × E findings appeared to be highly robust across informants, with similar effect sizes for the sG × E interactions across informants. These findings suggest that our significant sG × E interactions with a multi-informant latent index consisting of adolescent self-reports and both mother- and father-reports of parental psychological control or autonomy support were not driven by reports of one specific informant, but rather reflected a pattern of associations that was consistently present across informants.

## Discussion

In the present study, we aimed to conduct polygenic interaction (sG × E) analyses for adolescent social anxiety symptom development across 3 successive years that involved genetic variation in the oxytocin system and both constraining and facilitative aspects of parenting, that is, parental psychological control and autonomy support. To create polygenic components, we applied a novel analytical approach, that is, Principal Covariates Regression (De Jong and Kiers 1992; Vervloet et al. 2015), to 223 SNPs linked to the oxytocin system in a large sample of adolescents. Using this analytical technique, we identified one polygenic component—consisting of small contributions of many SNPs across multiple genes—that was strongly positively associated with adolescent social anxiety symptoms. Importantly, significant sG × E interactions suggested that higher scores on this polygenic oxytocin component were associated with higher initial levels of adolescent social anxiety in a context of higher parental psychological control or lower parental autonomy support. In contrast, lower scores on this polygenic oxytocin component were consistently associated with lower initial levels of social anxiety regardless of the parenting context. Collectively, these findings suggest that genetic variability in the oxytocin system interacts with both psychologically controlling and autonomy supporting parenting to predict adolescent social anxiety symptoms in line with the diathesis-stress model (Pluess 2015).

### Implications for Research on Social Anxiety Symptom Development in Adolescence

The present study expands substantially on current understanding of both genetic and environmental correlates of social anxiety symptom development in adolescence and their interactions. Regarding genetics, we identified five oxytocin-related polygenic components using Principal Covariates Regression. The oxytocin system has only recently been put forward as particularly relevant to symptoms of social anxiety (Gottschalk and Domschke 2016; Neumann and Slattery 2016) and has received some attention in earlier research on the genetic origins of social anxiety (e.g., Notzon et al. 2015; Thompson et al. 2011). Four of the identified polygenic components mainly represented non-random associations of alleles within a gene (i.e., LD structure) and thereby reflect information concerning population stratification (see Online Supplementary Material 1). Importantly, however, one polygenic component showed a strong positive association with adolescent social anxiety symptoms; a “multi-gene” risk component with very small contributions from multiple SNPs located in several genes across several chromosomes. This finding is in line with the prevailing notion of genetic vulnerability to psychopathology derived from GWAS studies (Visscher et al. 2012), which stresses the importance of polygenic approaches that consider many SNPs across different genes, as many genetic variants each appear to contribute only a small amount of risk.

While research on youths’ problem behavior and oxytocin-related genes other than *OXTR* is limited, the contribution of both the *PRLR* and *OXTR* genes to this polygenic component is in line with earlier findings that these genes are linked to restricted affiliative behaviors in autism (Yrigollen et al. 2008). Furthermore, *GABRA6*, the other gene that contributed to this polygenic component, has been associated with stress responsiveness, in social situations in particular, in past research (Frigerio et al. 2009; Uhart et al. 2004). As several genes were found to contribute to adolescent social anxiety symptoms, the oxytocin-related genetic basis of social anxiety seems complex. Oxytocin genes are assumed to confer some of their effects through intermediate physiological processes that have an impact on social interactions and social approach behavior, by affecting individuals’ responses to social stress (Bethlehem et al. 2014). However, being a statistical entity representing the combined genetic risk across multiple genetic markers like all polygenic scores, it is unknown through what biological (and potential psychological and social) mechanisms the captured genetic variability in oxytocin genes exactly affect a particular phenotype such as social anxiety. Also, additional research on the exact part of the genetic variability that is captured by the polygenic components uncovered in this study is clearly indicated. Furthermore, it should always be kept in mind that all genes and neurotransmitter systems interact as a whole in the human body and brain. We know that oxytocin is only part of a complex network of neurotransmitters and by focusing on some oxytocin-related genes, we look at a small part of these complex neurobiological circuits and from a specific angle only.

Regarding environmental influences, the current study revealed, in line with previous studies and theoretical models on the development of social anxiety symptoms (McLeod et al. 2007; Spence and Rapee 2016; Wong and Rapee 2015, 2016), that both constraining and facilitative aspects of the parental environment were associated with adolescent social anxiety symptoms. Some models suggest that when parents are highly psychologically controlling this may negatively affect adolescents’ feelings of self-efficacy and thereby be associated with elevated levels of anxiety, whereas parental encouragement of autonomy may positively affect adolescents’ feelings of self-efficacy and thereby be associated with lowered levels of anxiety (Chorpita and Barlow 1998; see also McLeod et al. 2007, for a brief discussion of different models). In our study, these associations between parenting and social anxiety were specifically found in relation to mean-level differences between adolescents, rather than changes in social anxiety symptoms over time. Importantly, parental psychological control and parental autonomy support—indicators of constraining and facilitative parenting, respectively, and assessed through a latent longitudinal multi-informant approach—only showed a modest negative correlation with one another across informants. This fits with conceptualizations of psychological control and autonomy support as independent parenting subdimensions rather than opposite ends of a common underlying continuum (Silk et al. 2003).

Regarding interactions between the polygenic components and the parental environment (sG × E), our findings suggested that the “multi-gene” polygenic oxytocin component interacted with both environmental factors to affect adolescent social anxiety symptoms. In line with the diathesis-stress framework, the combination of high genetic load and less adequate parenting (i.e., high psychological control or low autonomy support) represented a “dual risk” and was associated with the highest levels of adolescent social anxiety symptoms (Pluess 2015). Furthermore, our findings suggested that more adequate parenting, characterized by low constraining or high facilitative parenting, did not seem to protect adolescents’ much concerning their social anxiety symptom development in the presence of high genetic load. Low polygenic load, by contrast, was consistently associated with lower adolescent social anxiety symptoms regardless of the parenting context. Adolescents with low genetic risk thus appeared to be relatively unaffected by both positive and negative aspects of parenting, even though higher parental psychological control and lower parental autonomy support in itself were, as expected, significantly associated with adolescent social anxiety symptoms in our study.

So, whereas genetic risk and less adequate parenting appeared to be independently associated with adolescent social anxiety symptoms, the combined effect of these two types of risk factors should also be considered in sG × E analyses. Our polygenic sG × E findings are in line with (a) the developmental psychopathology framework (Cicchetti and Rogosch 2002), which recognizes that the development of all forms of psychopathology is affected by multiple risk as well as protective factors at multiple levels of analysis that interact with one another in complex ways over time, (b) current models of the development of social anxiety that include such interactions as a key element (e.g., Wong and Rapee 2015), and (c) earlier work on G × E interactions involving social anxiety that relied on a monogenic approach (e.g., Notzon et al. 2015; Reinelt et al. 2014; Thompson et al. 2011).

### Strengths, Limitations, and Directions for Future Research

The present study has several important strengths. First, by using polygenic components in our interaction analyses we implemented the latest recommendations in the research field, which involves a gradual shift from a focus on a single polymorphism in a single gene (i.e., candidate gene studies) to polygenic approaches (Belsky and Israel 2014; Dick et al. 2015). Second, by applying Principal Covariates Regression to genetic data, which is an established analytical approach in the field of psychology, we introduced researchers to a novel and versatile analytical tool for future polygenic sG × E studies. Third, by focusing on the relatively unexplored oxytocin system, which may have particular relevance for social anxiety symptoms compared to other psychopathological symptoms (Gottschalk and Domschke 2016; Neumann and Slattery 2016), we expanded on traditional approaches that rely on genetic variants in the dopamine or serotonin systems. Fourth, by taking a longitudinal approach, we took an important step in genetic interaction research in general, because most research so far has been cross-sectional in nature. Fifth and finally, by using a multi-informant latent index of parenting across 3 successive years as environmental factor in our sG × E analyses, we increased the assessment quality of the environmental factor (Wong et al. 2003). Furthermore, we considered both constraining and facilitative aspects of parenting in our interaction analyses. Whereas past research has predominantly focused on processes of risk, focusing on both positive and negative dimensions of parenting improves our understanding of processes of both resilience and risk, which are central processes in the developmental psychopathology perspective (Cicchetti and Rogosch 2002).

Still, our study should be considered in the light of some limitations, which may provide directions for future research. First, the usefulness of Principal Covariates Regression when creating polygenic components for use in sG × E studies is in need of replication in independent samples. Although several auxiliary analyses supported the validity, robustness, and specificity of our findings (see Online Supplementary Material 1), such analyses, valuable as they are, do not preclude the need for independent replications. Importantly, there is a growing need for replication and validation in the field of molecular genetics of complex traits in general (Dick et al. 2015; Halldorsdottir and Binder 2016). As sG × E studies become more complex in design, for example as they adopt a polygenic approach, focus on specific biological pathways, follow their participants over time, and use multi-informant assessments of the environment and/or the phenotype of interest, international and multidisciplinary collaboration in larger cohorts or consortia may become even more important in this field to be able to replicate and validate sG × E findings. At this moment, we have been unable to find studies with characteristics comparable to the key features of our own study to replicate and validate both the usefulness of Principal Covariates Regression for creating polygenic components and our significant sG × E findings.

Second, despite its strengths, this analysis technique has some weaknesses. SNP density within the genes, overall gene size, and—to a certain extent—the underlying LD structure are confounding factors in the type of analyses we conducted. It is no surprise, therefore, that the genes that contain the largest number of SNPs in our study (see Table S1 in Online Supplementary Material 1) strongly help to define the polygenic oxytocin components that we found. Or, in other words: genes with a small number of SNPs are less likely to define underlying polygenic components. Furthermore, it remains important to examine the composition of the identified genetic components in-depth to check it against known genetic interdependency of the polymorphisms (i.e., LD structure and ancestry) to avoid interpretation of confounding signals. In addition, our polygenic analyses build on the assumption that a genetic sensitivity to a certain phenotype can be detected in a first step, which moderates the association between environmental factors and this phenotype in a second step. This same assumption is also at the heart of the MPS and PRS approaches that our Principal Covariates Regression approach was intended to improve upon. Alternatively, some approaches exist that directly concentrate on significant G × E interactions at the gene or polymorphism level, such as *Genome-Wide by Environment Interaction Studies* (GWEIS; Dunn et al. 2016; Van Assche et al. 2017), but studies applying such approaches are still scarce.

Third, care should be taken not to overgeneralize our findings. The data were collected in a particular region of Western Europe on a relatively well-functioning community sample of adolescents with a relatively homogeneous ethnic background. It is as yet unclear whether our results can be extended to adolescents who live in other regions of the world, who have a more diverse socio-economic and ethnic background, and who are more diverse in functioning. Furthermore, interpretation of the associations and interactions observed is limited to the oxytocin system and the particular environmental variables included in our analyses. As indicated, other biological systems, most notably the serotonin and the dopamine systems (e.g., Enter et al. 2014; Gottschalk and Domschke 2016), also appear to play a role in social anxiety symptoms. However, the polygenic approach adopted in the current study, which involves a careful selection of genes within the oxytocin system and a subsequent selection of genetic variants within each of these genes, is generalizable and can easily be applied to any selection of genetic variants of interest. Similarly, other aspects of the parenting environment than the ones we examined, and parental overprotection in particular (Rubin et al. 2009; Wong and Rapee 2016), have also been linked with the etiology of social anxiety. Also, although we included adolescents’ sex, age, and living situation in our analyses as covariates of both the polygenic components and the development of adolescent social anxiety symptoms, there are many other unexamined potentially relevant covariates, also of parenting. Future research should expand on our study focusing on polymorphisms related to other neurotransmitters and other, or additional, parenting variables, as well as including other unexamined potentially relevant covariates, particularly of the parenting variable(s) under study. On a related note, we were unable to control for potential effects of pubertal status or the menstrual cycle of females on adolescent social anxiety symptom development (Reardon et al. 2009; Van Veen et al. 2009), which would be important potential covariates for future research to include.

Fourth and finally, an important challenge for the research field is to incorporate longitudinal assessments of the environmental factor included in sG × E interactions in addition to longitudinal assessments of the phenotype of interest, because we know that environmental factors including parenting are dynamic in nature and may thereby show change over time. Also, it is important to realize that all significant findings, including our own sG × E findings, are mere statistical results that are in need of an explanation in terms of their underlying mechanisms. Only if those mechanisms are understood at the molecular level and linked to increased risk for social anxiety can we further increase our understanding and interpretation of polygenic component-by-environment interactions (Halldorsdottir and Binder 2016).

### Conclusion

The present study represents an important step forward in the creation of polygenic (oxytocin) scores based on many different SNPs located in different genes and toward the inclusion of these polygenic scores in sG × E interaction research on adolescent (social anxiety symptom) development. Collectively, the findings suggest that polygenic risk in the oxytocin system interacts with both psychologically controlling and autonomy supporting parenting to predict adolescent social anxiety symptom development. Specifically, in line with a diathesis-stress framework, the findings suggested that higher polygenic risk combined with either low facilitative parenting or high constraining parenting predicted higher levels of social anxiety symptoms over time. Principal Covariates Regression, the statistical technique employed in this study, may be a useful and versatile analytical tool for future polygenic sG × E interaction research.

## Electronic supplementary material

ESM 1 (DOCX 443 kb)

## Footnotes

1 Using the method described by Benjamini and Hochberg (1995) to control for false discovery rate, our corrected level of significance was calculated as *p* < 0.01625 (instead of *p* < 0.05) for both main sG × E interaction analyses. Relying on this corrected *p*-value level, our findings concerning main effects of polygenic component 1 and parenting and our significant sG × E interactions remained significant at *p* < 0.01625 and were thus robust to corrections for false discovery rate.

## References

[CR1] American Psychiatric Association (2013). Diagnostic and statistical manual of mental disorders.

[CR2] Anderson CA, Pettersson FH, Clarke GM, Cardon LR, Morris AP, Zondervan KT (2010). Data quality control in genetic case-control association studies. Nature Protocols.

[CR3] Bair E, Hastie T, Paul D, Tibshirani R (2006). Prediction by supervised principal components. Journal of the American Statistical Association.

[CR4] Barber, B. K. (2002). Regulation as a multicultural concept and construct for adolescent health and development. Unpublished manuscript.

[CR5] Barlow DH (2002). Anxiety and its disorders: The nature and treatment of anxiety and panic.

[CR6] Barrett JC, Fry B, Maller J, Daly MJ (2005). Haploview: Analysis and visualization of LD and haplotype maps. Bioinformatics.

[CR7] Belsky DW, Israel S (2014). Integrating genetics and social science: Genetic risk scores. Biodemography and Social Biology.

[CR8] Benjamini Y, Hochberg Y (1995). Controlling the false discovery rate: A practical and powerful approach to multiple testing. Journal of the Royal Statistical Society. Series B (Methodological).

[CR9] Bethlehem RAI, Baron-Cohen S, Van Honk J, Auyeung B, Bos PA (2014). The oxytocin paradox. Frontiers in Behavioral Neuroscience.

[CR10] Bijttebier P, Beck I, Claes L, Vandereycken W (2009). Gray's reinforcement sensitivity theory as a framework for research on personality-psychopathology associations. Clinical Psychology Review.

[CR11] Chorpita BF, Barlow DH (1998). The development of anxiety: The role of control in the early environment. Psychological Bulletin.

[CR12] Cicchetti D, Rogosch FA (2002). A developmental psychopathology perspective on adolescence. Journal of Consulting and Clinical Psychology.

[CR13] Cohen J (1992). A power primer. Psychological Bulletin.

[CR14] De Jong S, Kiers HAL (1992). Principal Covariates Regression. Part I. Theory. Chemometrics and Intelligent Laboratory Systems.

[CR15] De Leeuw CA, Mooij JM, Heskes T, Posthuma D (2015). MAGMA: Generalized gene-set analysis of GWAS data. PLoS Computational Biology.

[CR16] Dick DM, Agrawal A, Keller MC, Adkins A, Aliev F, Monroe S (2015). Candidate gene-environment interaction research: Reflections and recommendations. Perspectives on Psychological Science.

[CR17] Dunn EC, Wiste A, Radmanesh F, Almli LM, Gogarten SM, Sofer T (2016). Genome-wide association study (GWAS) and genome-wide by environment interaction study (GWEIS) of depressive symptoms in African American and Hispanic/Latina women. Depression and Anxiety.

[CR18] Ebstein RP, Knafo A, Mankuta D, Chew SH, Lai PS (2012). The contributions of oxytocin and vasopressin pathway genes to human behavior. Hormones and Behavior.

[CR19] Enter D, Colzato LS, Roelofs K (2014). Dopamine transporter polymorphisms affect social approach - avoidance tendencies. Genes, Brain and Behavior.

[CR20] Frigerio A, Ceppi E, Rusconi M, Giorda R, Raggi ME, Fearon P (2009). The role played by the interaction between genetic factors and attachment in the stress response in infancy. Journal of Child Psychology and Psychiatry.

[CR21] Gottschalk MG, Domschke K (2016). Novel developments in genetic and epigenetic mechanisms of anxiety. Current Opinion in Psychiatry.

[CR22] La Greca, A. M., & Lopez, N. (1998). Social anxiety among adolescents: Linkages with peer relations and friendships. *Journal of Abnormal Child Psychology, 26*, 83–94. 10.1023/A:1022684520514. 10.1023/a:1022684520514 9634131

[CR23] Grolnick WS, Ryan RM, Deci EL (1991). Inner resources for school achievement: Motivational mediators of children’s perceptions of their parents. Journal of Educational Psychology.

[CR24] Halldorsdottir T, Binder E (2016). Gene x environment interactions: From molecular mechanisms to behavior. Annual Review of Psychology.

[CR25] Illumina (2010). *Infinium HD methylation assay protocol guide*. Retrieved from http://www.illumina.com

[CR26] Illumina (2011). *Infinium LCG assay experienced user card, manual protocol*. Retrieved from http://www.illumina.com

[CR27] Janssens A, Goossens L, Van den Noortgate W, Colpin H, Verschueren K, Van Leeuwen K (2015). Parents’ and adolescents’ perspectives on parenting: Evaluating conceptual structure, measurement invariance, and criterion validity. Assessment.

[CR28] Kessler RC, Avenevoli S, Costello EJ, Georgiades K, Green JG, Gruber MJ (2012). Prevalence, persistence, and sociodemographic correlates of DSM-IV disorders in the National Comorbidity Survey Replication-Adolescent Supplement. Archives of General Psychiatry.

[CR29] Kingery JN, Erdley CA, Marshall KC, Whitaker KG, Reuter TR (2010). Peer experiences of anxious and socially withdrawn youth: An integrative review of the developmental and clinical literature. Clinical Child and Family Psychology Review.

[CR30] McLeod BD, Wood JJ, Weisz JR (2007). Examining the association between parenting and childhood anxiety: A meta-analysis. Clinical Psychology Review.

[CR31] Muthén LK, Muthén BO (1998). Mplus user’s guide.

[CR32] Nelemans, S. A., Meeus, W. H. J., Branje, S. J. T., Van Leeuwen, K., Colpin, H., Verschueren, K., & Goossens, L. (2017). Social anxiety scale for adolescents (SAS-A) short form: Longitudinal measurement invariance in two community samples of youth. *Assessment*. Advance online publication. 10.1177/1073191116685808 10.1177/1073191116685808 28052690

[CR33] Neumann, I. D., & Slattery, D. A. (2016). Oxytocin in general anxiety and social fear: A translational approach. *Biological Psychiatry, 79*, 213–221. 10.1016/j.biopsych.2015.06.004. 10.1016/j.biopsych.2015.06.004 26208744

[CR34] Nikolova YS, Ferrell RE, Manuck SB, Hariri AR (2011). Multilocus genetic profile for dopamine signaling predicts ventral striatum reactivity. Neuropsychopharmacology.

[CR35] Notzon S, Domschke K, Holitschke K, Ziegler C, Arolt V, Pauli P (2015). Attachment style and oxytocin receptor gene variation interact in influencing social anxiety. World Journal of Biological Psychiatry.

[CR36] Otowa T, Hek K, Lee M, Byrne EM, Mirza SS, Nivard MG (2016). Meta-analysis of genome-wide association studies of anxiety disorders. Molecular Psychiatry.

[CR37] Peyrot WJ, Milaneschi Y, Abdellaoui A, Sullivan PF, Hottenga JJ, Boomsma DI, Penninx (2014). Effect of polygenic risk scores on depression in childhood trauma. British Journal of Psychiatry.

[CR38] Pluess M (2015). Individual differences in environmental sensitivity. Child Development Perspectives.

[CR39] Purcell SM, Wray NR, Stone JL, Visscher PM, O’Donovan MC, Sullivan PF (2009). Common polygenic variation contributes to risk of schizophrenia and bipolar disorder. Nature.

[CR40] Rapee RM, Vasey MW, Dadds MR (2001). The development of generalized anxiety. The developmental psychopathology of anxiety.

[CR41] Reardon LE, Leen-Feldner EW, Hayward C (2009). A critical review of the empirical literature on the relation between anxiety and puberty. Clinical Psychology Review.

[CR42] Reinelt E, Aldinger M, Stopsack M, Schwahn C, John U, Baumeister SE, Grabe HJ, Barnow S (2014). High social support buffers the effects of 5-HTTLPR genotypes within social anxiety disorder. European Archives of Psychiatry and Clinical Neuroscience.

[CR43] Rubin KH, Coplan RJ, Bowker JC (2009). Social withdrawal in childhood. Annual Review of Psychology.

[CR44] Salvatore JE, Aliev F, Bucholz K, Agrawal A, Hesselbrock V, Hesselbrock M (2015). Polygenic risk for externalizing disorders: Gene-by-development and gene-by-environment effects in adolescents and young adults. Clinical Psychological Science.

[CR45] Scaini S, Belotti R, Ogliari A (2014). Genetic and environmental contributions to social anxiety across different ages: A meta-analytic approach to twin data. Journal of Anxiety Disorders.

[CR46] Schneier FR, Heckelman LR, Garfinkel R, Campeas R, Fallon B, Gitow A (1994). Functional impairment in social phobia. Journal of Clinical Psychiatry.

[CR47] Siess J, Blechert J, Schmitz J (2014). Psychophysiological arousal and biased perception of bodily anxiety symptoms in socially anxious children and adolescents: A systematic review. European Child & Adolescent Psychiatry.

[CR48] Silk JS, Morris AS, Kanaya T, Steinberg L (2003). Psychological control and autonomy granting: Opposite ends of a continuum or distinct constructs?. Journal of Research on Adolescence.

[CR49] Spence SH, Rapee RM (2016). The etiology of social anxiety disorder: An evidence-based model. Behaviour Research and Therapy.

[CR50] Szklarczyk D, Franceschini A, Kuhn M, Simonovic M, Roth A, Minguez P, Doerks T, Stark M, Muller J, Bork P, Jensen LJ, Von Mering C (2011). The STRING database in 2011: Functional interaction networks of proteins, globally integrated and scored. Nucleic Acids Research.

[CR51] Thompson RJ, Parker KJ, Hallmayer JF, Waugh CE, Gotlib IH (2011). Oxytocin receptor gene polymorphism (rs2254298) interacts with familial risk for psychopathology to predict symptoms of depression and anxiety in adolescent girls. Psychoneuroendocrinology.

[CR52] Uhart M, McCaul ME, Oswald LM, Choi L, Wand GS (2004). GABRA6 gene polymorphism and an attenuated stress response. Molecular Psychiatry.

[CR53] Van Assche, E., Moons, T., Cinar, O., Viechtbauer, W., Oldehinkel, A. J., Van Leeuwen, K., … Van Winkel, R. (2017). Gene-based interaction analysis shows GABAergic genes interacting with parenting in adolescent depressive symptoms. *Journal of Child Psychology and Psychiatry*. Advance online publication. 10.1111/jcpp.12766, 58, 1301, 1309. 10.1111/jcpp.12766 28660714

[CR54] Van Veen JF, Jonker BW, Van Vliet IM, Zitman FG (2009). The effects of female reproductive hormones in generalized social anxiety disorder. International Journal of Psychiatry in Medicine.

[CR55] Vervloet M, Kiers HAL, Van den Noortgate W, Ceulemans E (2015). PCovR: An R package for principal covariates regression. Journal of Statistical Software.

[CR56] Visscher PM, Brown MA, McCarthy MI, Yang J (2012). Five years of GWAS discovery. American Journal of Human Genetics.

[CR57] Vrshek-Schallhorn S, Stroud CB, Mineka S, Zinbarg R, Adam EK, Redei EE (2015). Additive genetic risk from five serotonin system polymorphisms interacts with interpersonal stress to predict depression. Journal of Abnormal Psychology.

[CR58] Wong, Q. J. J., & Rapee, R. M. (2015). The developmental psychopathology of social anxiety and phobia in adolescents. In K. Ranta, A. M. La Greca, L. J. Garcia-Lopez, & M. Marttunen (Eds.), *Social anxiety and phobia in adolescents: Development, manifestation and intervention strategies*. 10.1007/978-3-319-16703-9_2.

[CR59] Wong QJJ, Rapee RM (2016). The etiology and maintenance of social anxiety disorder: A synthesis of complimentary theoretical models and formulation of a new integrated model. Journal of Affective Disorders.

[CR60] Wong MY, Day NE, Luan JA, Chan KP, Wareham NJ (2003). The detection of gene-environment interaction for continuous traits: Should we deal with measurement error by bigger studies or better measurement?. International Journal of Epidemiology.

[CR61] Yrigollen CM, Han SS, Kochetkova A, Babitz T, Chang JT, Volkmar FR (2008). Genes controlling affiliative behavior as candidate genes for autism. Biological Psychiatry.

