# Supplementary material for: Parenting Interacts with Oxytocin Polymorphisms to Predict Adolescent Social Anxiety Symptom Development: A Novel Polygenic Approach
Source: J Abnorm Child Psychol. 2018 Apr 26;47(7):1107–20. doi: 10.1007/s10802-018-0432-8 (PMC6599763; doi:10.1007/s10802-018-0432-8)
Supplement: Supplementary file 1 — (DOCX 443 kb) [file 10802_2018_432_MOESM1_ESM.docx]

**Online Supplementary Material 1**

**Article title:** Parenting Interacts With Oxytocin Polymorphisms to Predict Adolescent Social Anxiety Symptom Development: A Novel Polygenic Approach

**Journal name:** Journal of Abnormal Child Psychology

**Doi:** 10.1007/s10802-018-0432-8

**Author names and affiliation:** Stefanie A. Nelemans^a,b^, Evelien van Assche^c,d^, Patricia Bijttebier^a^, Hilde Colpin^a^, Karla van Leeuwen^e^, Karine Verschueren^a^, Stephan Claes^c,d^, Wim van den Noortgate^f^, & Luc Goossens^a^

^a^Research Unit School Psychology and Development in Context, KU Leuven, Belgium; ^b^Research Center Adolescent Development, Utrecht University, the Netherlands;  ^c^GRASP-Research Group, Department of Neuroscience, KU Leuven, Belgium; ^d^University Psychiatric Center KU Leuven, Belgium; ^e^Parenting and Special Education Research Group, KU Leuven, Belgium; ^f^Methodology of Educational Sciences Research Group, KU Leuven, Belgium.

**E-mail address of corresponding author:** s.a.nelemans@uu.nl

**Procedure Genetic Data and Quality Control.**

Population data was obtained from the 1,000 Genomes Project (The 1000 Genomes Project Consortium, 2012), an online database of genetic variation in several populations across the globe. Within this database, data from three Caucasian populations were used, the central Utah European descendants (CUE), Great Britain (GBR), and Tuscany Italy (TSI), as these were thought to resemble the targeted population most closely. Samples were prepared for genotyping at the Center for Human Genetics in Leuven, Belgium. For extraction, 2 mL of the saliva samples were prepared using Chemagen MSM I (Perkin Elmer^®^) in combination with Chemagic DNA Saliva2k kit special by the protocol “Chemagic DNA Saliva 2k H24 EB 13 mL prefilling VD120328.che”. Following these steps, concentration was measured using Victor^3^ (Perking Elmer). The total number of SNPs selected in our procedure was 6,325. A total of 7,043 SNPs (i.e., 6,325 from which a selection of 718 SNPs that were genotyped in duplicate) was analyzed using an Illumina Infinium iSelect Custom BeadChip. All SNPs were manually reviewed using the GenomeStudio Genotyping Module (Illumina, 2008, 2010b).

The protocols of Anderson et al. (2010) and Purcell et al. (2009) were used for quality control, which resulted in a total of 5,052 SNPs. Of these 5,052 SNPs, 223 SNPs were located at 14 genes of our predefined pathway of oxytocin-related genes. Furthermore, following the protocols of Anderson et al. (2010) and Purcell et al. (2009) for quality control, data from 1,031 participants was deemed of high quality (93.4% of all genotyped participants; 48.8% girls; *M*_age_ T_1_= 13.79, *SD*_age_ T_1_ = 0.94). Specifically, data from 7 participants were removed because they had a genetic call rate less than 95%, data from 2 participants were removed because they had incompatible levels of X-chromosome heterozygosity (i.e., PLINK check-sex test), and, finally, in order to obtain a population sample, data from one member of 63 sibling pairs were randomly removed based on IBD (i.e., PLINK rel-check). SNPs were coded additively.

**Auxiliary Analyses.**

Several additional analyses were conducted to examine the robustness, validity, and specificity of our polygenic findings.

**Robustness analyses.** To examine the robustness of our findings, we examined how several factors affected the polygenic components or associations between the polygenic components and adolescent social anxiety symptoms. For these analyses, we used maximum likelihood estimation with standard errors and chi-square robust to non-normality (i.e., MLR estimator; Muthén & Muthén, 1998-2015).

***Polygenic components and adolescents’ age, family structure, and sex*.** Adolescents’ age was virtually not associated with adolescents’ scores on the five polygenic components, *r*s = -.06 to .08. There were no significant differences in the five polygenic scores depending on adolescents’ family structure, *F*(5, 964) = 1.79, *p* = .11, or sex, *F*(5, 964) = 0.56, *p* = .73.

***Including adolescents’ age, sex, and family structure as covariates*.** Including adolescents’ age, sex, and family structure as potential covariates for adolescent social anxiety symptoms in the regression analysis did not substantially affect associations between the five polygenic components and adolescent social anxiety symptoms, even though adolescents’ sex was a significant predictor of adolescent social anxiety symptoms, β = .26, 95% CI [.15, .37], *p* < .001 (i.e., girls reported higher levels of social anxiety symptoms than boys). Specifically, in the analyses including these covariates, the one polygenic component with substantive interpretation in relation to adolescent social anxiety symptoms still showed a strongly positively association, β = .49, *p* < .001. Finally, adolescents’ sex did not significantly moderate any of the associations between the polygenic component scores and adolescent social anxiety symptoms, βs = -.03 to .06, *p*s > .11.

***SNP subsets*.** Randomly deleting 1%, 5%, or 10% of the oxytocin SNPs (i.e., 2, 11, or 22 SNPs out of the 223 oxytocin SNPs, respectively) from the PCovR analyses did not substantially affect the predictive values of the five polygenic components on adolescent social anxiety symptoms nor the composition of the five polygenic components. Specifically, deleting 1%, 5%, or 10% of the oxytocin SNPs of the oxytocin SNPs still resulted in one polygenic component that was strongly significantly associated with adolescent social anxiety symptoms, β = .49, *p* < .001, β = .46, *p* < .001, and β = .45, *p* < .001, respectively, while the other four resulting components appeared to still strongly reflect the underlying LD structure.

**Interpretation of Polygenic Components.** For more in-depth interpretation of our polygenic components, we checked the composition of the five polygenic components in-depth.

***Genetic composition of polygenic components*.** Researchers with a strong background in genetics could be concerned that the polygenic components identified by PCovR are but a mere reflection of the underlying genetic structure or non-random association of alleles at different loci (i.e., linkage disequilibrium or LD). Or, in less technical language, some researchers could worry that the components are mainly based on the simple fact that SNPs that are located close together in the human genome often tend to be strongly correlated. By combining information from several methods (including SNAP, PLINK, and Hapmap), we examined the genetic composition of the five polygenic scores to identify how strongly they reflected the underlying LD structure.

Since LD is an important source of interdependency between SNPs, we expected that if α is closer to 1 in PCovR, the components can largely be explained by the underlying LD structure. On the left side of Figure 2 in the main body of the manuscript at α values close to 1 (assigning a greater role to reduction of the 223 oxytocin SNPs than prediction of adolescent social anxiety symptoms), four polygenic components were found. As the explained variance in adolescent social anxiety symptoms was close to zero, this was a first suggestion that these four components could be largely explained by the underlying LD structure (i.e., only explaining variance in the SNPs). As anticipated, combined information from several methods indeed suggested that the composition of these four polygenic components appeared to be a close reflection of the underlying LD structure. Results are graphically displayed in a heatmap in Figure S2.

However, at α values from 0.88 to 0.80 (assigning a somewhat greater role to prediction of adolescent social anxiety symptoms), a fifth polygenic component was found and the proportion of explained variance in adolescent social anxiety symptoms greatly increased (see Figure 2 in the main body of the manuscript). This suggests that in addition to the four polygenic components that strongly reflected the underlying genetic structure, this fifth polygenic component would likely be of particular interest to adolescent social anxiety symptoms. Importantly, this polygenic component that was most strongly associated with adolescent social anxiety symptoms was spread across many genes and combined information from several methods suggested that this component was more than just a mere reflection of the underlying LD structure.

***Population stratification*.** Researchers with a strong background in genetics could be concerned about potential confounding effects of population stratification on our results. Specifically, these researchers could be concerned that the polygenic PCovR components are inaccurate because of our lack of correction for broader genomic architecture related to ancestral origin (i.e., population stratification). Or, in less technical language, researchers could worry that the identified polygenic components are not controlled for individual differences in genetic variants due to systematic differences in ancestry.

Using Principal Components Analysis (PCA) in PLINK, a technique that is traditionally used within a genetic context to correct for population stratification (Price et al., 2006), we used our selection of 223 oxytocin-related SNPs for all 1,031 participants to identify ancestrally informative dimensions. In our study, the first PCA dimension clearly explained most of the genetic variation, as indicated by the eigenvalues of all 20 PCA dimensions (see Table S3). A clear inflection point was visible in the scree plot from the first to the second dimension. Therefore, we considered this first component to most likely capture ancestrally informative information, but also considered the second PCA dimension in all subsequent analyses.

First, Pearson’s correlations suggested that one of the polygenic PCovR components was moderately to strongly positively associated with the two ancestrally informative PCA dimensions. Specifically, this polygenic PCovR component (Component 4 in Figure S2, which was not significantly associated with adolescent social anxiety symptoms) was significantly associated with both ancestrally informative PCA dimensions, *r*s = .28-.50. Importantly, regression analysis suggested that 36% of the variance in this polygenic PCovR component could be explained by the two ancestrally informative PCA dimensions. These ancestrally informative PCA dimensions only explained 0.29 to 2.66% of the variance in any of the other three polygenic PCovR components with no substantive interpretation in relation to adolescent social anxiety symptoms (i.e., Components 2, 3, and 5 in Figure S2). These findings suggest that individual differences in genetic variants due to systematic differences in ancestry one of the identified PCovR components (i.e., Component 4) appeared to capture.

Second, we included the two ancestrally-informative dimensions in the sG×E interaction analyses as covariates of the polygenic oxytocin components as well as initial levels and change in adolescent social anxiety symptom development across 3 successive years to examine potential effects on our sG×E findings. Importantly, findings concerning interactions between the polygenic oxytocin component with substantive interpretation in relation to adolescent social anxiety symptoms and both parental psychological control and parental autonomy support were exactly the same including population stratification as covariate as the findings not including population stratification as covariate (reported on pp. 18-19 of the main body of the manuscript). Specifically, results still suggested a significant interaction between the polygenic oxytocin component that was substantively associated with adolescent social anxiety symptoms (consisting of small contributions of many SNPs across multiple genes) and our latent index of parental psychological control, β = .09, 95% CI [.03, .16], *p* = .005, as well as a our latent index of parental autonomy support, β = -.11, 95% CI [-.04, -.18], *p* = .001, on initial/intercept levels of adolescent social anxiety. In addition, we found a significant interaction between this polygenic component and our latent index of parental psychological control, β = -.14, 95% CI [-.03, -.25], *p* = .015, as well as a marginally significant interaction with our latent index of parental autonomy support, β = .10, 95% CI [-.02, .21], *p* = .088, on change in levels of adolescent social anxiety across 3 successive years.

Third and final, we included the two ancestrally-informative dimensions as covariates in our PCovR analyses in order to control for genetic ancestry, and hence correct for population stratification, while creating the polygenic oxytocin components. First, we explored PCovR results for different α values ranging from 0 to 1 in steps of 0.10 to get an idea of the optimal α value. As large changes in explained variance appeared to occur between α values of .70 and 1.00, we conducted a more in-depth exploration of PCovR results across this range of values in steps of 0.01. An α value of .83 appeared to show the best balance between reduction of the oxytocin SNPs, controlling for population stratification, and prediction of adolescent social anxiety symptoms. In this PCovR solution, one polygenic component was again strongly positively associated with adolescent social anxiety symptoms, β = .49, 95% CI [.44, .54], *p* < .001. Interestingly, correcting for population stratification made a substantive interpretation of this polygenic component clearer as this component was now characterized by 41 SNPs with strong effect sizes (i.e., ≥ .35 in absolute value). Most SNPs were located in the *GABRA6* (*n*  = 7), the *OXTR* (*n* = 13), and the *PRLR* (*n* = 13) genes, in line with results for this particular component without correcting for population stratification in the PCovR analyses. Importantly, findings concerning the sG×E interaction analyses with both parental psychological control and parental autonomy support in relation to initial levels and change in these levels across 3 successive years using the polygenic oxytocin components corrected for population stratification were similar to the results not correcting for population stratification (reported on pp.18-19 of the main body of the manuscript and in Table 1). See Table S4 for detailed results.

In sum, findings suggest that 1) individual differences in genetic variants due to systematic differences in ancestry are captured to some degree into some of the PCovR components with no substantive interpretation in relation to adolescent social anxiety symptom development, 2) the polygenic oxytocin components resulting from PCovR appear to contribute significant additional variance in adolescent social anxiety symptom development correcting for population stratification, and 3) including the ancestrally-informative dimensions in the PCovR analyses to correct the polygenic components for population stratification does not affect findings in the sG×E interaction analyses concerning both parental psychological control and parental autonomy support in relation to initial levels and change in adolescent social anxiety symptoms across 3 successive years.

**Specificity Analyses.** To examine the specificity of our findings, we examined associations between the polygenic components and relevant forms of adolescent psychosocial functioning other than social anxiety symptoms.

***Polygenic components and adolescent broader psychological functioning*.** To examine if the polygenic components were more strongly associated with adolescent social anxiety symptoms than with adolescent depressive symptoms, feelings of loneliness, levels of neuroticism, and externalizing symptoms, we regressed all these variables on the five polygenic components.

Depressive symptoms were assessed with the Center for Epidemiologic Studies Depression scale (CES-D; Radloff, 1991). A sample item reads “I felt depressed”. All 20 items were rated on a 4-point Likert-type scale, ranging from 0 (*rarely or none of the time*) to 3 (*most or all of the time*). Internal consistency of the CES-D was good (Cronbach's α = .91). Higher scores reflect higher levels of depressive symptoms. Feelings of loneliness were assessed with the 12-item loneliness in peer relations subscale of the Loneliness and Aloneness Scale for Children and Adolescents (LACA; Marcoen, Goossens, & Caes, 1987). A sample item reads “I feel isolated from other people”. All items were rated on a 4-point Likert-type scale, ranging from 1 (*never*) to 4 (*often*). Internal consistency of the peer-related loneliness subscale was good (Cronbach's α = .91). Higher scores reflect higher levels of loneliness. Levels of neuroticism were assessed with the 6-item neuroticism subscale of the Quick Big Five (QBF; Vermulst & Gerris, 2005). Participants had to rate themselves on six adjectives (i.e., anxious, fearful, fretful, high-strung, irritable, and nervous) on a 7-point Likert-type scale, ranging from 1 (*completely untrue*) to 7 (*completely true*). Internal consistency of the neuroticism subscale was good (Cronbach's α = .79). Higher scores reflect higher levels of neuroticism. Externalizing symptoms were assessed with the 31-item externalizing subscale of the Youth Self-Report (YSR; Achenbach & Rescorla, 2001). A sample item reads “I physically attack people”. All items were rated on a 3-point Likert-type scale, ranging from 0 (*not true*) to 2 (*very true or often true*). Internal consistency of the externalizing subscale was good (Cronbach's α = .83). Higher scores reflect higher levels of externalizing symptoms.

In the analyses, we controlled for symptom overlap between all phenotypes by including all possible correlations among the outcome variables. For the analyses, we used maximum likelihood estimation with standard errors and chi-square robust to non-normality (i.e., MLR estimator; Muthén & Muthén, 1998-2015). Results are graphically displayed in Figure S3.

The first polygenic component appeared to be strongly associated with adolescent social anxiety symptoms, β = .49, *p* < .001, moderately associated with adolescent depressive symptoms, β = .17, *p* < .001, feelings of loneliness, β = .24, *p* < .001, and levels of neuroticism, β = .25, *p* < .001, and weakly associated with adolescent externalizing symptoms, β = .08, *p* = .036. Significant Wald tests indicated that the first polygenic component was associated more strongly with adolescent social anxiety symptoms than with any of the other outcomes, Wald χ^2^s (1) > 8.25, *p*s < .005. Furthermore, the first polygenic component was associated more strongly with adolescent social anxiety symptoms, depressive symptoms, feelings of loneliness, and levels of neuroticism than with adolescent externalizing symptoms, Wald χ^2^s (1) > 14.92, *p*s < .001. Whereas the first polygenic component thus appeared to be associated most strongly with adolescent social anxiety symptoms, attesting to the validity of our analyses, this component also appeared to reflect a broader vulnerability factor, in particular for several forms of internalizing symptoms or traits but also, to a certain degree, for externalizing symptoms.

The second component appeared to be moderately associated with adolescent social anxiety symptoms, β = -.19, *p* < .001, depressive symptoms, β = -.11, *p* < .001, feelings of loneliness, β = -12, *p* < .001, and levels of neuroticism, β = -.10, *p* = .002, but not significantly associated with adolescent externalizing symptoms, *p* = .21. Significant Wald tests indicated that the second polygenic component was associated more strongly with adolescent social anxiety symptoms than with any of the other outcomes, Wald χ^2^s (1) > 12.94, *p*s < .001, except for levels of neuroticism, Wald χ^2^ (1) = 1.25, *p* = .26. Whereas the second component thus appeared to be associated most strongly with adolescent social anxiety symptoms, attesting to the validity of our analyses, this component also appeared to reflect a protective factor for a somewhat broader internalizing construct or trait, but not for externalizing problems. Finally, the third component appeared to be solely associated with adolescent social anxiety symptoms, albeit weakly, β = .06, *p* = .037. For the second and third component, we of course have to keep in mind that they can be largely explained by the underlying LD structure. Therefore, it is likely that associations can be driven by only one or two SNPs in these components.

**Sensitivity analyses.** A multi-informant latent index consisting of adolescent self-reports and both mother- and father-reports of parental psychological control or autonomy support across 3 successive years was included as environmental factor in our main sG×E interaction analyses to increase the assessment quality of environmental exposure (Wong, Day, Luan, Chan, & Wareham, 2003). However, to examine robustness of our sG×E findings across informants we conducted additional separate sG×E interaction analyses for adolescents, mothers, and fathers. In addition, we conducted a sG×E interaction analysis with a multi-informant latent index consisting of both mother- and father-reports combined (to make adolescent- and parent-reports of psychological control or autonomy support comparable, since adolescents reported on psychological control or autonomy support of their parents in general, while mothers and fathers reported on their own psychological control or autonomy support towards the adolescent).

Importantly, our sG×E findings appeared to be highly robust across informants, with similar effect sizes for the sG×E interactions across informants (even though not all interactions were statistically significant at *p* < .05). Please see an overview of the sG×E findings for the different informants in Table S5. These findings suggest that our significant sG×E interactions with a multi-informant latent index consisting of adolescent self-reports and both mother- and father-reports of parental psychological control or autonomy support were not driven by reports of one specific informant, but rather reflected a pattern of associations that was consistently present across informants.

**References**

Achenbach, T. M., & Rescorla, L. (2001). *ASEBA school-age forms & profiles*. Burlington, VT: University of Vermont, Research Center for Children, Youth, & Families.

Anderson, C. A., Pettersson, F. H., Clarke, G. M., Cardon, L. R., Morris, A. P., & Zondervan, K. T. (2010). Data quality control in genetic case-control association studies. *Nature Protocols, 5*, 1564-1573. doi:10.1038/nprot.2010.116

Illumina (2008). *GenomeStudio genotyping module v1.0 user guide.* Retrieved from http://www.illumina.com

Illumina (2010). *Interpreting Infinium^®^ assay data for whole-genome structural variation.* Retrieved from http://www.illumina.com

Marcoen, A., Goossens, L., & Caes, P. (1987). Loneliness in pre- through late adolescence: Exploring the contributions of a multidimensional approach. *Journal of Youth and Adolescence, 16*, 561-577. doi:10.1007/BF02138821

Muthén, L. K. & Muthén, B. O. (1998-2015). *Mplus user’s guide* (7^th^ ed.). Los Angeles, CA: Muthén & Muthén.

Price, A. L., Patterson, N. J., Plenge, R. M., Weinblatt, M. E., Shadick, N. A., & Reich, D. (2006). Principal components analysis corrects for stratification in genome-wide association studies. *Nature Genetics, 38*, 904-909. doi:10.1038/ng1847

Purcell, S. M., Wray, N. R., Stone, J. L., Visscher, P. M., O’Donovan, M. C., Sullivan, P. F., … Sklar, P. (2009). Common polygenic variation contributes to risk of schizophrenia and bipolar disorder. *Nature, 460*, 748-752. doi:10.1038/nature08185

Radloff, L. S. (1991). The use of the Center for Epidemiologic Studies Depression scale in adolescents and young adults. *Journal of Youth and Adolescence, 20*, 149-166.

doi:10.1007/BF01537606

Roberts, R. E., & Sobhan, M. (1992). Symptoms of depression in adolescence: A comparison of Anglo, African, and Hispanic Americans. *Journal of Youth and Adolescence, 21*, 639-651. doi:10.1007/BF01538736

The 1000 Genomes Project Consortium. (2012). An integrated map of genetic variation from 1,092 human genomes. *Nature, 491*, 56-65. doi:10.1038/nature11632

Vermulst, A. A., & Gerris, J. R. M. (2005). *QBF: Quick Big Five persoonlijkheidstest handleiding* [Quick Big Five personality test manual]. Leeuwarden, the Netherlands: LDC Publications.

Wong, M. Y., Day, N. E., Luan, J. A., Chan, K. P., & Wareham, N. J. (2003). The detection of gene-environment interaction for continuous traits: Should we deal with measurement error by bigger studies or better measurement? *International Journal of Epidemiology, 32*, 51-57. doi:10.1093/ije/dyg002

Table S1

*Distribution of 223 Oxytocin Single Nucleotide Polymorphisms (SNPs) According to Gene and Chromosome Location*

| Gene | Protein | Chromosome | Number of SNPs |
| --- | --- | --- | --- |
| *OXTR* | Oxytocin receptor | 3 | 51 |
| *PRLR* | Prolactin receptor | 5 | 47 |
| *GABRA6* | Gamma-aminobutyric acid receptor alpha-6 subunit | 5 | 21 |
| *VEGFA* | Vascular endothelial growth factor | 6 | 17 |
| *CALCRL* | Calcitonin receptor-like receptor | 2 | 14 |
| *AVPR1B* | Arginine vasopressin receptor 1B | 1 | 12 |
| *GNRHR* | Gonadotropin-releasing hormone receptor | 4 | 11 |
| *TCF20* | Transcription factor 20 | 22 | 10 |
| *CGA* | Glycoprotein hormones alpha polypeptide | 6 | 8 |
| *OXT* | Oxytocin | 20 | 8 |
| *AVP* | Arginine vasopressin | 20 | 7 |
| *EN2* | Homeobox protein engrailed-2 | 7 | 7 |
| *PRL* | Prolactin | 6 | 7 |
| *ARNT2* | Aryl hydrocarbon receptor nuclear translocator 2 | 15 | 3 |

Table S2

*Summary of Means and Standard Deviations for all Study Variables Across 3 Years*

| Variable | *M* | *SD* |
| --- | --- | --- |
| Social anxiety symptoms T_1_ | 2.40 | 0.79 |
| Social anxiety symptoms T_2_ | 2.55 | 0.81 |
| Social anxiety symptoms T_3_ | 2.53 | 0.80 |
| Adolescent-reported psychological control T_1_ | 1.97 | 0.65 |
| Adolescent-reported psychological control T_2_ | 2.00 | 0.67 |
| Adolescent-reported psychological control T_3_ | 1.95 | 0.63 |
| Adolescent-reported autonomy support T_1_ | 3.88 | 0.64 |
| Adolescent-reported autonomy support T_2_ | 3.85 | 0.62 |
| Adolescent-reported autonomy support T_3_ | 3.89 | 0.58 |
| Mother-reported psychological control T_1_ | 1.79 | 0.51 |
| Mother-reported psychological control T_2_ | 1.77 | 0.48 |
| Mother-reported psychological control T_3_ | 1.78 | 0.51 |
| Mother-reported autonomy support T_1_ | 4.18 | 0.47 |
| Mother-reported autonomy support T_2_ | 4.19 | 0.44 |
| Mother-reported autonomy support T_3_ | 4.22 | 0.43 |
| Father-reported psychological control T_1_ | 1.92 | 0.51 |
| Father-reported psychological control T_2_ | 1.89 | 0.51 |
| Father-reported psychological control T_3_ | 1.88 | 0.53 |
| Father-reported autonomy support T_1_ | 3.99 | 0.53 |
| Father-reported autonomy support T_2_ | 4.00 | 0.50 |
| Father-reported autonomy support T_3_ | 4.00 | 0.50 |

Table S3

*Overview of Eigenvalues of all 20 Dimensions Resulting from PLINK Principal Components Analysis* (*N*_SNPs_ = 223, *N*_sample_ = 1,031)

| PCA dimension | Eigenvalue |
| --- | --- |
| 1 | 685.41 |
| 2 | 44.63 |
| 3 | 32.74 |
| 4 | 31.38 |
| 5 | 26.27 |
| 6 | 25.27 |
| 7 | 22.70 |
| 8 | 20.74 |
| 9 | 20.07 |
| 10 | 19.62 |
| 11 | 17.98 |
| 12 | 17.42 |
| 13 | 16.21 |
| 14 | 15.29 |
| 15 | 14.75 |
| 16 | 14.30 |
| 17 | 12.93 |
| 18 | 12.70 |
| 19 | 12.22 |
| 20 | 11.34 |

Table S4

*Structural Equation Models Predicting Adolescent Social Anxiety Symptom Development Across 3 Successive Years With Polygenic Components Corrected for Population Stratification (N = 973)*

|  | Parental psychological control | | | |  | Parental autonomy support | | | |
| --- | --- | --- | --- | --- | --- | --- | --- | --- | --- |
|  | Intercept | | Linear slope | |  | Intercept | | Linear slope | |
| Predictor | *b (SE)* | β | *b (SE)* | β |  | *b (SE)* | β | *b (SE)* | β |
| Parenting | 0.16 (0.03)^***^ | .26 | 0.01 (0.02) | .04 |  | -0.08 (0.03)^**^ | -.13 | -0.00 | -.01 |
| Polygenic C1 | 0.20 (0.04)^***^ | .32 | 0.01 (0.03) | .02 |  | 0.20 (0.04)^***^ | .33 | 0.01 (0.03) | .03 |
| Polygenic C2 | -0.11 (0.04)^*^ | -.17 | 0.00 (0.03) | .01 |  | -0.11 (0.04)^**^ | -.18 | 0.00 (0.03) | .01 |
| Polygenic C3 | -0.02 (0.04) | -.03 | 0.03 (0.03) | .09 |  | -0.03 (0.04) | -.04 | 0.03 (0.03) | .10 |
| Polygenic C4 | -0.01 (0.04) | -.01 | -0.01 (0.02) | -.03 |  | -0.01 (0.04) | -.01 | -0.01 (0.02) | -.03 |
| Polygenic C5 | 0.08 (0.04)^*^ | .13 | -0.05 (0.02)^*^ | -.18 |  | 0.08 (0.04)^*^ | .13 | -0.05 (0.02) ^*^ | -.18 |
| Parenting × C1 | 0.06 (0.02)^**^ | .09 | -0.04 (0.02)^*^ | -.14 |  | -0.07 (0.02)^**^ | -.11 | 0.02 (0.02) | .08 |
| Sex | 0.23 (0.04)^***^ | .37 | 0.06 (0.03)^*^ | .20 |  | 0.22 (0.04)^***^ | .36 | 0.06 (0.03)^*^ | .20 |
| Age | -0.01 (0.02) | -.01 | -0.01 (0.01) | -.05 |  | 0.01 (0.02) | .01 | -0.01 (0.01) | -.01 |
| Family structure | -0.08 (0.05) | -.13 | 0.04 (0.03) | .13 |  | -0.08 (0.05) | -.14 | 0.04 (0.03) | .13 |

*Note.* C1-C5 represent the five polygenic oxytocin components resulting from the PCovR analysis, including population stratification. Sex was coded 0 for boys and 1 for girls and family structure was coded 0 for living in intact two-parent families and 1 for other family structure. ^*^ *p* ≤ .05. ^**^ *p* ≤ .01. ^***^ *p* ≤ .001.

Table S5

*Summary of sG×E Findings for Different Informants on Parental Psychological Control and Parental Autonomy Support Predicting Adolescent Social Anxiety Symptom Development Across 3 Successive Years (N = 973)*

|  | Parental psychological control | | | |  | Parental autonomy support | | | |  |
| --- | --- | --- | --- | --- | --- | --- | --- | --- | --- | --- |
|  | Intercept | | Linear slope | |  | Intercept | | Linear slope | |  |
| sG×E analyses | *b (SE)* | β | *b (SE)* | β |  | *b (SE)* | β | *b (SE)* | β | |
| Main sG×E analyses | 0.06 (0.02)^**^ | .09 | -0.04 (0.02)^**^ | -.16 |  | -0.07 (0.02)^***^ | -.10 | 0.03 (0.02)^†^ | .10 | |
| Adolescent-report only | 0.05 (0.02)^*^ | .07 | -0.04 (0.02)^*^ | -.14 |  | -0.06 (0.02)^**^ | -.09 | 0.02 (0.02) | .08 | |
| Mother-report only | 0.03 (0.03) | .05 | -0.03 (0.02)^†^ | -.12 |  | -0.05 (0.03)^†^ | -.07 | 0.00 (0.02) | .01 | |
| Father-report only | 0.05 (0.03)^*^ | .08 | -0.04 (0.02)^†^ | -.13 |  | -0.05 (0.03)^†^ | -.07 | 0.03 (0.02) | .10 | |
| Parent-reports combined (in latent index) | 0.04 (0.02)^†^ | .06 | -0.04 (0.02)^*^ | -.13 |  | -0.05 (0.02)^*^ | -.08 | 0.02 (0.02) | .08 | |

*Note*. ^†^*p* ≤ .10. ^*^*p* ≤ .05. ^**^*p* ≤ .01. ^***^*p* ≤ .001.

*
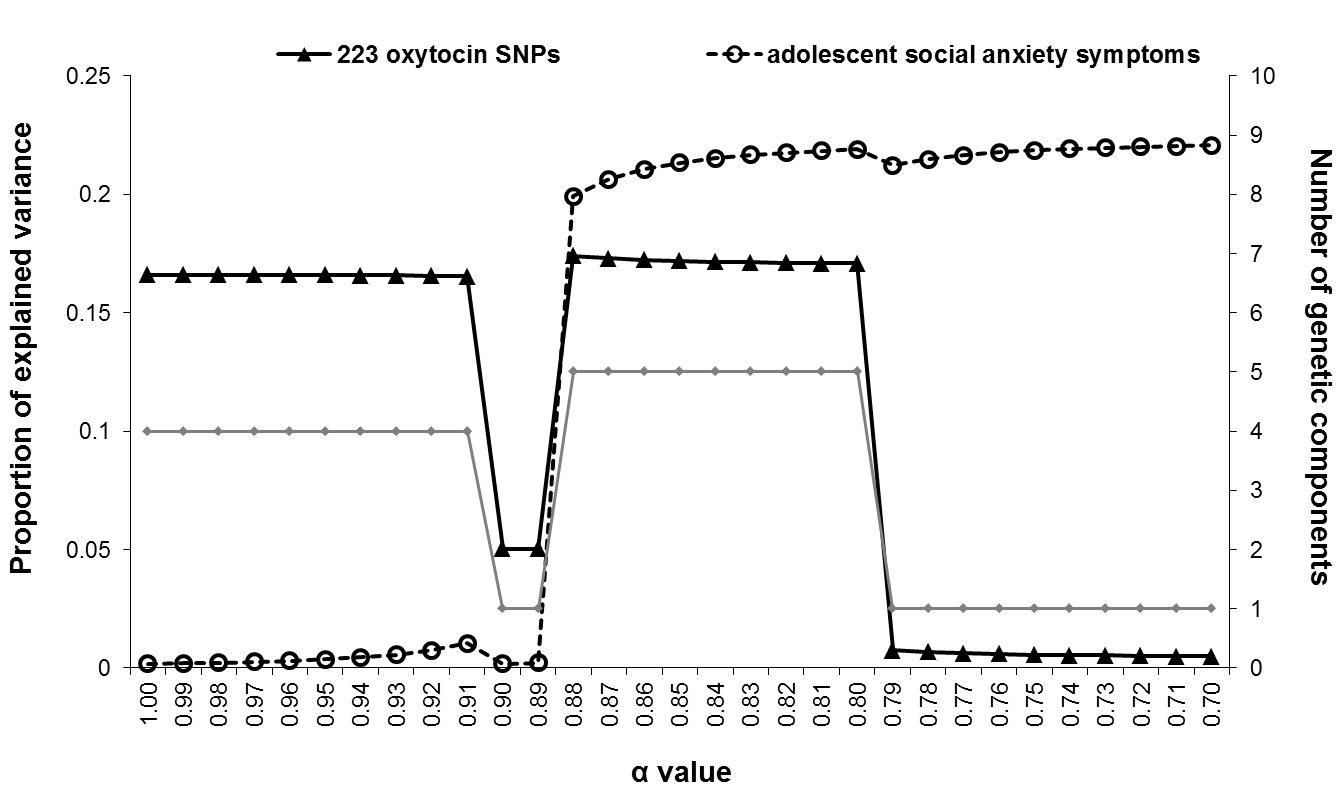
*

*Figure S1.* Graphic representation of the proportion of explained variance in the 223 oxytocin SNPs (black solid line with solid triangle markers) and adolescent social anxiety symptoms (i.e., black dotted line with open circle markers) for all α values from 0.70 to 1.00 by steps of 0.01. Higher α values (i.e., closer to 1) assign a greater role to reduction of the 223 oxytocin SNPs when constructing the components and lower α values (i.e., closer to 0) assign a greater role to prediction of adolescent social anxiety symptoms when constructing the components. The total number of genetic components that are extracted for each PCovR solution is plotted on the right Y-axis and is visualized by the grey solid line.


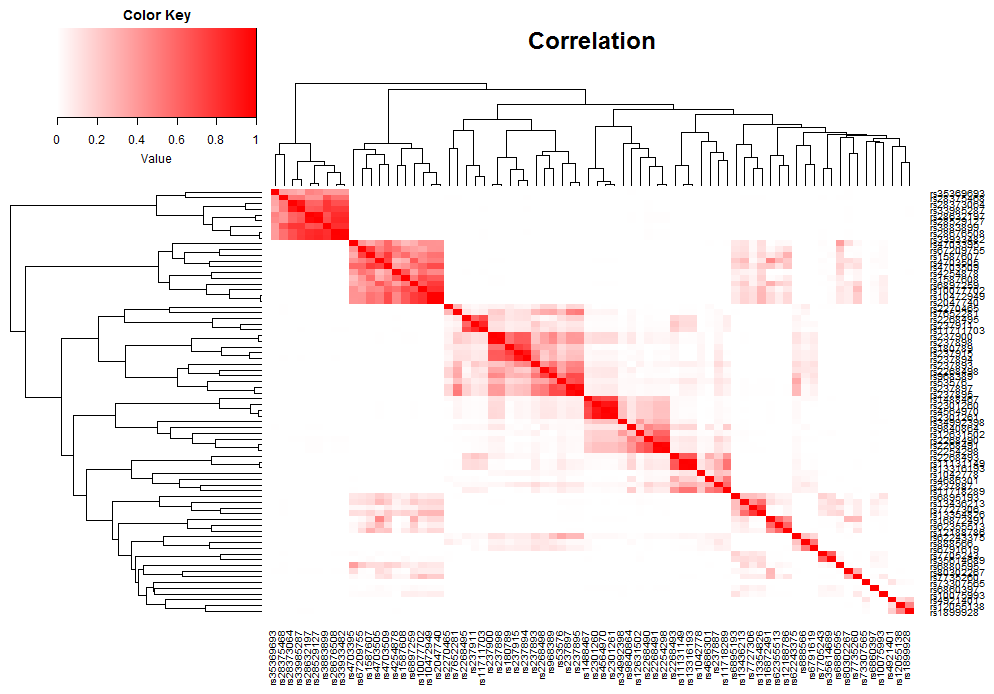


Component 5

Component 4

Component 3

Component 2

*Figure S2*. Heatmap of all SNPs with factor loadings showing medium effect sizes (i.e., ≥ .15 in absolute value) for the first polygenic component and large effect sizes (i.e., ≥ .35 in absolute value) for all other genetic components. Correlations among SNPs are indicated in red color, with stronger correlations among SNPs being represented by a more intense red color.

*
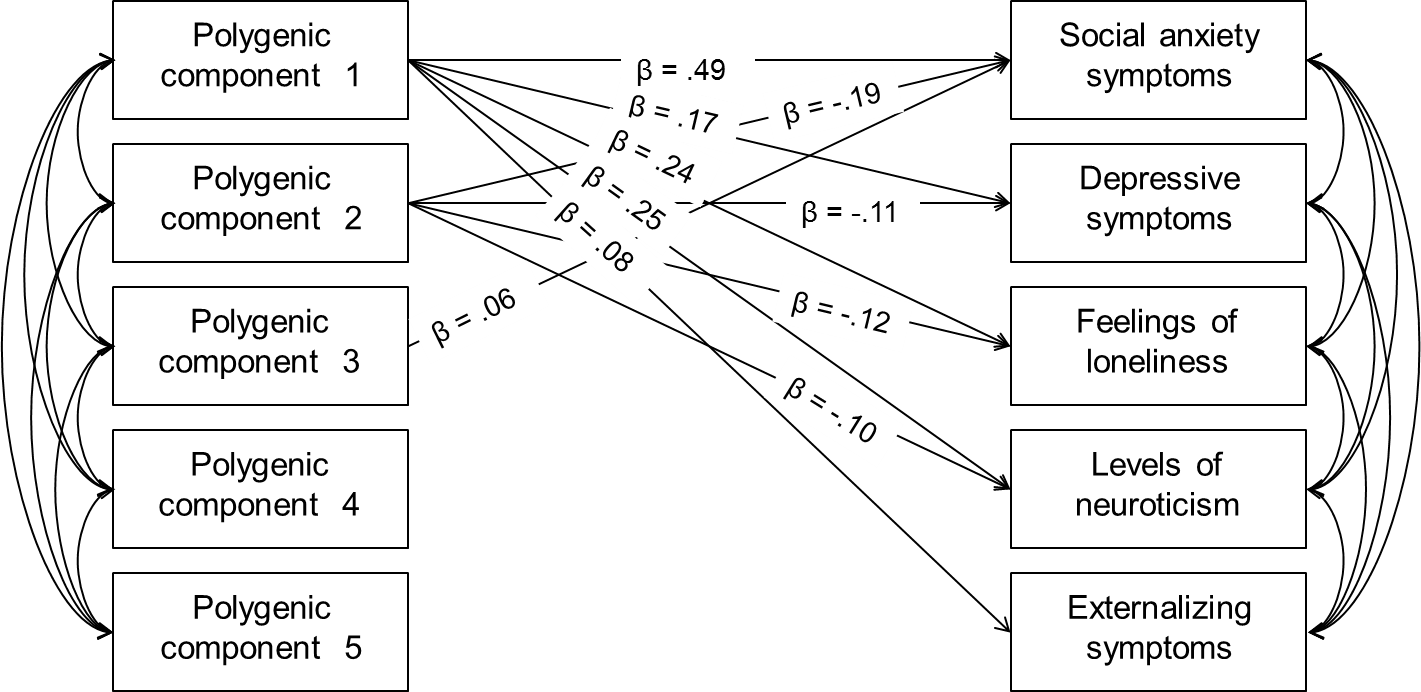
*

*Figure S3*. Graphical representation of standardized associations (βs) between the five polygenic oxytocin components that resulted from PCovR analysis with adolescent social anxiety symptoms and adolescent social anxiety symptoms, depressive symptoms, feelings of loneliness, levels of neuroticism, and externalizing symptoms. Only statistically significant (*p* < .05) associations are shown.
